# Supplementary material for: The Evolution of Effort-Reward Imbalance in Workers during the COVID-19 Pandemic in France—An Observational Study in More than 8000 Workers
Source: Int J Environ Res Public Health. 2022 Jul 26;19(15):9113. doi: 10.3390/ijerph19159113 (PMC9331729; doi:10.3390/ijerph19159113)
Supplement: Supplementary file 1 [file ijerph-19-09113-s001.zip › ijerph-1779957-supplementary.pdf]

**Supplementary Material:**

**Supplementary Table S1:** Summary of the second-order interactions between variables in the final GEE model on the full population.

**Supplementary Table S2:** Summary of the second-order interactions between variables in the sensitivity analysis of the GEE model performed on the subgroup in whom the Effort Reward Imbalance score was collected for the pre-pandemic, lockdown 1 and lockdown 2 periods.

**Supplementary Table S3:** Exploratory analysis of factors associated with ERI score as a quantitative variable for each period of interest, using linear bivariate and multivariate analysis.

**Supplementary Figure S1:** Sensitivity analysis of the GEE models, using the subgroup of respondents in whom the ERI was collected for the pre-pandemic, lockdown 1 and lockdown 2 periods.

**Supplementary Table S1.** Summary of the second-order interactions between variables in the final GEE model on the full population.

| **Variable Interaction Term** | **Coefficient** | **95% CI** | ***p*–Value** |
|---|---|---|---|
| occupation [medical] * Nb Hours per Week [>50] | −1.42 | −2.66 to −0.18 | 0.025 |
| sex [Female] * number of children [4 or more] | −10.36 | −17.92 to −2.80 | 0.007 |
| sex [Female] * Nb Hours per Week [30–40] | 1.12 | 0.06 to 2.18 | 0.038 |
| age [35–45] * number of children [1] | −7.42 | −12.59 to −2.25 | 0.005 |
| age [above 65] * Nb Hours per Week [30–40] | −4.39 | −7.68 to −1.09 | 0.009 |
| age [above 65] * Nb Hours per Week [40–50] | −2.88 | −5.60 to −0.17 | 0.037 |
| marital status [other] * number of children [3] | −14.92 | −28.95 to −0.88 | 0.037 |
| Nb Hours per Week [>50] * period | −0.34 | −0.57 to −0.11 | 0.004 |
| Nb Hours per Week [40–50] * period | −0.28 | −0.49 to −0.06 | 0.011 |

**Supplementary Table S2.** Summary of the second−order interactions between variables in the sensitivity analysis of the GEE model performed on the subgroup in whom the Effort Reward Imbalance score was collected for the pre−pandemic, lockdown 1 and lockdown 2 periods.

| **Variable Interaction Term** | **Coefficient** | **95% CI** | ***p*−Value** |
|---|---|---|---|
| occupation2 [paramedical] * age [above 65] | 22.17 | 3.00 to 41.34 | 0.023 |
| sex [Female] * age [35–45] | −8.59 | −16.87 to −0.31 | 0.042 |
| age [above 65] * marital status [as couple] | −29.75 | −52.05 to −7.46 | 0.009 |
| age [above 65] * Nb hours per week [30–40] | −22.86 | −45.41 to −0.31 | 0.047 |
| age [above 65] * Nb hours per week [40–50] | −18.11 | −36.16 to −0.06 | 0.049 |
| marital status [other] * number of children [1] | −29.81 | −47.95 to −11.66 | 0.001 |

**Supplementary Table S3.** Exploratory analysis of factors associated with ERI score as a quantitative variable for each period of interest, using bivariate and multivariate analysis.

| Variables | Pre–pandemic Period | | | | Lockdown 1 | | | | Lockdown 2 | | | |
|---|---|---|---|---|---|---|---|---|---|---|---|---|
| | **Bivariate** | **Multivariate** | | | **Bivariate** | **Multivariate** | | | **Bivariate** | **Multivariate** | | |
| | ***p*–Value** | **Estimate** | **95% CI** | ***p*–Value** | ***p*–Value** | **Estimate** | **95% CI** | ***p*–Value** | ***p*–Value** | **Estimate** | **95% CI** | ***p*–Value** |
| Age (reference [35–45]) | **< 0.001** | | | | **0.08** | | | | 0.48 | | | |
| Age [45–55] | | −0.86 | [−3.3 : 1.6] | 0.49 | | 0.6 | [−1.3 : 2.5] | 0.5 | | – | – | – |
| Age [55–65] | | 4.9 | [2.2 : 7.6] | **< 0.001** | | 2.2 | [−0.05 : 4.5] | 0.05 | | – | – | – |
| Age [> 65] | | 11.1 | [5.1 : 17] | **< 0.001** | | −3.4 | [−8.6 : 1.8] | 0.2 | | – | – | – |
| Age [< 35] | | 0.9 | [−1.9 : 3.8] | 0.52 | | 2.2 | [0.1 : 4.3] | **0.04** | | – | – | – |
| Marital Status (Reference : Single) | 0.9 | | | | 0.9 | | | | 0.6 | | | |
| Marital Status : Couple | | – | – | – | | – | – | – | | – | – | – |
| Marital Stauts : Other | | – | – | – | | – | – | – | | – | – | – |
| Number of children (reference : 0) | **0.03** | | | | **0.049** | | | | 0.75 | | | |
| Number of children : 1 | | 1.14 | [−1.8 : 4.1] | 0.45 | | −0.9 | [−3.1 : 1.3] | 0.4 | | – | – | – |
| Number of children : 2 | | −0.02 | [−2.6 : 2.6] | 0.99 | | −0.1 | [−2.1 : 1.8] | 0.9 | | – | – | – |
| Number of children : 3 | | 0.03 | [−3.2 : 3.3] | 0.98 | | 0.6 | [−1.9 : 3.2] | 0.6 | | – | – | – |
| Number of children : ≥ 4 | | 2.71 | [−1.9 : 7.4] | 0.25 | | 3.3 | [−0.6 : 7.3] | 0.1 | | – | – | – |
| Sex (reference : Male) | **0.02** | | | | 0.16 | | | | 0.17 | | | |
| Sex : Female | | 1.06 | [−1.1 : 3.3] | 0.34 | | – | – | | | – | – | – |
| Occupation (reference : Non-Healthcare) | **< 0.001** | | | | **< 0.001** | | | | **0.02** | | | |
| Occupation : Medical | | 0.73 | [−1.6 : 3.1] | 0.54 | | 4.1 | [2 : 6.3] | **< 0.001** | | 4.9 | [0.6 : 9.2] | **0.03** |
| Occupation : Paramedical | | −2.86 | [−5.5 :−0.2] | **0.03** | | 0.8 | [−1.1 : 2.7] | 0.4 | | −2.2 | [−5.7 : 1.3] | 0.22 |
| Weekly working hours (reference : [30–40]) | **< 0.001** | | | | **< 0.001** | | | | 0.67 | | | |
| Weekly working hours : < 30 | | 2.24 | [−0.8 : 5.3] | 0.14 | | −1.5 | [−3.6 : 0.5] | 0.1 | | – | – | – |
| Weekly working hours : > 50 | | 4.36 | [1.5 : 7.2] | **< 0.01** | | 4.4 | [2 : 6.9] | **< 0.001** | | – | – | – |
| Weekly working hours : 40–50 | | 2.89 | [0.4 : 5.4] | **0.02** | | 1.9 | [−1 : 3.8] | 0.07 | | – | – | – |
| **Performance metrics** | | | | | | | | | | | | |
| RSE (Residual Standard Error) | – | 0.42 | | | – | 0.55 | | | – | 0.51 | | |
| Adjusted $R^2$ | – | 0.02 | | | – | 0.01 | | | – | 0.003 | | |

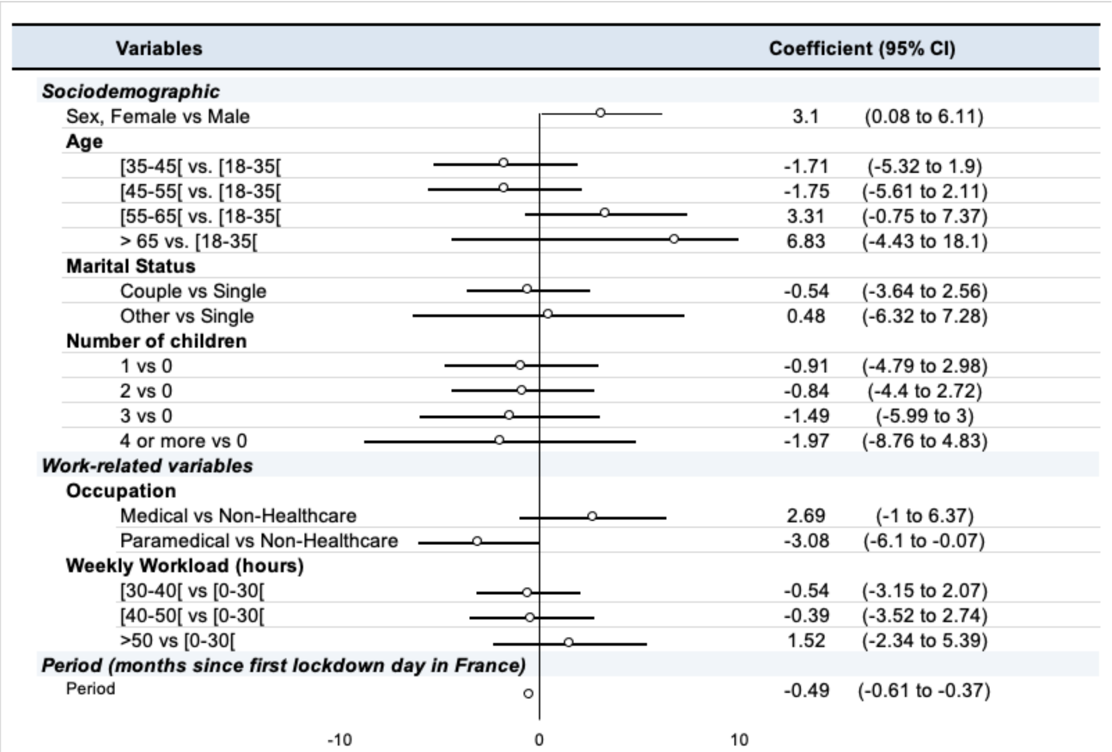


**Supplementary Figure S1.** Summary of sensitivity analysis for the GEE model with exchangeable covariance structure (GEE−ex), performed on the subgroup in whom the Effort Reward Imbalance score was collected for the pre−pandemic, lockdown 1 and lockdown 2 periods.
